# XGB-DrugPred: computational prediction of druggable proteins using eXtreme gradient boosting and optimized features set

**DOI:** 10.1038/s41598-022-09484-3

**Published:** 2022-04-01

**Authors:** Rahu Sikander, Ali Ghulam, Farman Ali

**Affiliations:** 1 School of Computer Science and Technology, Xidian University, Xi’an, 710071 China; 2 Computerization and Network Section, Sindh Agriculture University, Tandojam, Pakistan; 3 School of Computer Science and Engineering, Nanjing University of Science and Technology, Nanjing, China

**Keywords:** Computational biology and bioinformatics, Molecular biology

## Abstract

Accurate identification of drug-targets in human body has great significance for designing novel drugs. Compared with traditional experimental methods, prediction of drug-targets via machine learning algorithms has enhanced the attention of many researchers due to fast and accurate prediction. In this study, we propose a machine learning-based method, namely XGB-DrugPred for accurate prediction of druggable proteins. The features from primary protein sequences are extracted by group dipeptide composition, reduced amino acid alphabet, and novel encoder pseudo amino acid composition segmentation. To select the best feature set, eXtreme Gradient Boosting-recursive feature elimination is implemented. The best feature set is provided to eXtreme Gradient Boosting (XGB), Random Forest, and Extremely Randomized Tree classifiers for model training and prediction. The performance of these classifiers is evaluated by tenfold cross-validation. The empirical results show that XGB-based predictor achieves the best results compared with other classifiers and existing methods in the literature.

## Introduction

The analysis of Human Genome Project can provide the opportunity for pharmacologists to design novel drugs with specific targets in disease. Due to complicated system biology of most diseases, the newly developed drugs are not only limited but their effect in treating disease is also poor^1^. Thus, it is indispensable to design unique and effective drugs for diseases. A protein that interacts with drug is called druggable protein. Mostly druggable proteins are classified into nuclear receptors and functional proteins. It has been reported by past studies that druggable proteins are closely involved in cancers, cardiovascular, immune system, and other chronic diseases^2^.

Recently, the emergence of computerized algorithms and modeling in biology has made great progress in drug discovery^3^. These computational approaches are developed to determine the drug-disease interaction and how drugs affect targets in diseases. The computational approaches in drug-target discovery are based on either statistical or machine learning models. For instance, several researchers have implemented the secondary structure information of proteins and functional domains for analysis of drug-target interaction^4^. Some researchers adopted 3D structural features to analyze whether drug can bind on the surface of a protein^5–7^. However, due to the non-availability of 3D structure information of all proteins in the databank, their application is limited^8,9^.

With the passage of time, machine learning models were established for prediction of drug-target proteins. These models presented amino acid composition and di-peptide composition for identification of targets^10–12^. Sequence-based calculations of amino acid/protein features are useful because it can be computed easily and mostly predict protein function accurately. In this connection, many researchers employed different feature extraction methods and classification algorithms for prediction of drug-target interaction. Yu et al. used PROFEAT software to explore 1080 feature vector with support vector machine and random forest^13^. Chen et al. integrated basic features of protein using sequence, secondary, and subcellular localization as well as support vector machine for prediction of drug-targets in ion channels^12^. Han et al. yielded overall accuracy of 84% by implementing support vector machine with tenfold cross-validation^14^. Jamali et al. fused amino acid composition, dipeptide composition with physicochemical features and performed the classification by neural network^15^. The authors achieved 92.1% accuracy with fivefold cross-validation. Yamanishi et al. investigated protein sequence similarity, structural similarity, and protein interaction networks. The model was trained by nuclear regression to identify drug-target using genomic and chemical space^16^. Bleakley et al. introduced bipartite local model (BLM) approach to improve the prediction accuracy^17^. In other efforts, Lin et al. first extracted features by dipeptide composition, reduced sequence algorithms, and PseAAC and then integrated^2^. The best features were selected by genetic algorithm. The optimal features were fed into Bagging-SVM ensemble classifier and achieved an accuracy of 93.78%. Furthermore, Chen X et al. discussed the new evaluation validation framework and the formulation of drug-target interactions prediction problem by more realistic regression formulation based on quantitative bioactivity data^18^.

**Figure 1:**
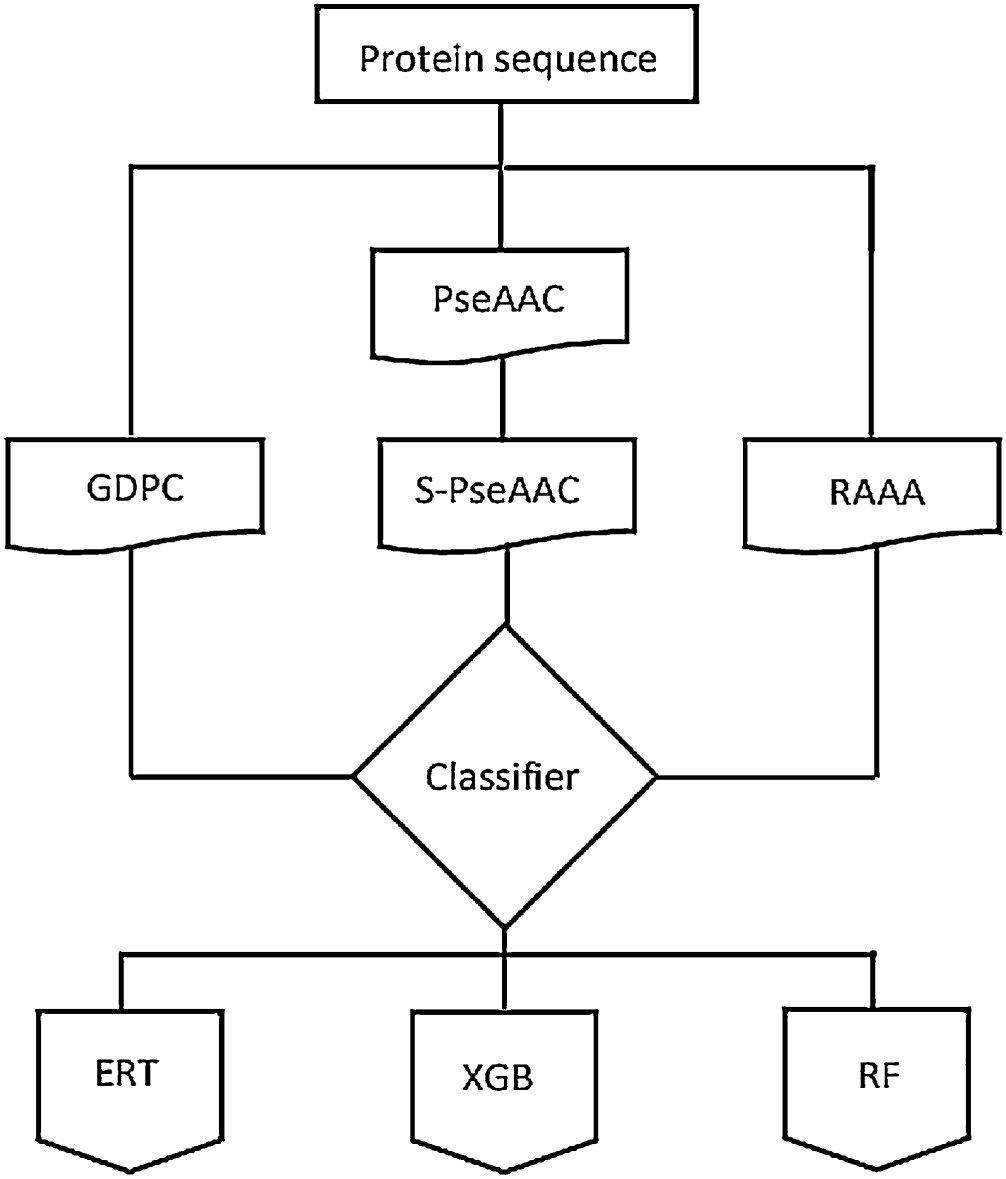
Schematic view of the proposed model.

All the above-cited methods have shown great contribution in prediction of drug-target interaction, however, each predictor has its limitation. For example, structure-based methods are expensive and limited applications due to the unavailability of structural information of all proteins in the databanks^19–21^. Most existing predictors have used conventional feature extraction methods such as amino acid composition, dipeptide composition, and position specific scoring matrix, however, these approaches do not effectively explore the important features. Moreover, integrated form of these features produces high dimensional vector space that leads to redundant features as well as high computational time. Due to the crucial role of druggable proteins in diverse cellular and biological processes, it is needed to design a computational method that can efficiently predict druggable proteins. To cover the above limitations of the existing predictors, we present a promising predictor, called XGB-DrugPred. In this study, the features are explored by group dipeptide composition, reduced amino acid alphabet, and novel encoder pseudo amino acid segmentation (S-PseAAC). To obtain multi-perspective feature vector, we concatenated all features to make a super set. A novel feature selection algorithm namely eXtreme Gradient Boosting-recursive feature elimination is adopted for selection of best features. The optimal features are provided to eXtreme Gradient Boosting, Random Forest, and Extremely Randomized Tree. Each classifier is trained and prediction performance is assessed by tenfold CV with five parameters i.e., accuracy, sensitivity, specificity, F-measure, and Mathew’s correlation coefficient. Among all models, XGB-based model has not only secured the best performance but also achieved the highest results compared with existing predictors in the literature. The schematic view of the proposed model has shown in Fig. 1.

## Material and methods

### Dataset

A benchmark dataset usually contains positive samples (proteins that can interact with drugs) and negative samples (proteins that cannot interact with drugs). For a fair comparison with existing methods, we used the dataset constructed by Jamali et al.^15^. The 1611 druggable proteins were retrieved from DrugBank database as explained by past study. Among these proteins, similar sequences in terms of features and content were removed using CD-HIT tool. The final positive samples set contains 1224 druggable proteins. Similarly, the negative samples set is constructed by combining datasets proposed by Bakheet et al.^22^ and Li et al.^10^. Initially, these sequences were collected from Swiss-Prot database. After eliminating the similar sequences, the remaining sequences were 1319 non-druggable proteins. The final benchmark dataset contains 1611 druggable proteins and 1224 non-druggable proteins.

### Feature encoding methods

#### Grouped dipeptide composition

Grouped Dipeptide Composition (GDPC) feature descriptor is an advance form of the DPC encoder. In this approach, amino acids are grouped into five classes using their physicochemical properties i.e., aromatic group (W, Y, and F), positive charge group (H, K, and R), aliphatic group (A, I, M, G, L, and V), uncharged group (C, T, P, S, Q, and N), and negative charged group (D, E, and G)^23^. The feature space of the GDPC can be formulated as:

1 $$f\left(m,n\right)=\frac{{T}_{mn}}{T-1}, m,n\in (G1, G2, G3, G4, G5)$$

Here, ${T}_{mn}$ is the frequency of dipeptide indicated by amino acid of groups $m \mathrm{and} n$ while $T$ represents the length of peptide or protein sequence.

#### Reduced amino acid alphabet

Feature extraction is a key step in the construction of a computational method. However, high dimension feature vector may cause several issues such as high time complexity and overfitting. To deal with these problems, we applied reduced amino acid alphabet (RAAA) as feature extraction approach. RAAA uses the physiochemical properties and grouped the amino acid residues into smaller groups which not only reduced the complexity of protein sequences but also explore the structural local regions and structural similarity^24^. We clustered the amino acids into five groups i.e., (C(5), C(8), C(9), C(11), and C(13) according to the procedure defined by Etchebest et al.^25^. which is explained in the following equation:

2 $$\left\{\begin{array}{c}C\left(5\right)=(G;IVFYW;ALMEQRK;P;NDHSTC)\\ C\left(8\right)=(G;IV;FYW;ALM;EQRK;P;ND;HSTC)\\ C\left(9\right)=G;IV;FYW;ALM;EQRK;P;ND;HS;TC)\\ C\left(11\right)=G;IV;FYW;A;LM;EQRK;P;ND;HS;T;C)\\ C\left(13\right)=G;IV;FYW;A;L;M;E;QRK;P;ND;HS;T;C)\end{array}\right.$$

In $C\left(j\right)$, $j$ shows the number of clusters in each group and the clusters are separated by semicolon.

#### Pseudo amino acid composition segmentation

A protein sequence contains 20 amino acids. To compute the occurrence frequency of these amino acids in a protein sequence, Amino Acid Composition (AAC) was introduced^26^. However, AAC avoids the sequence order information and correlation factors. To cover these deficiencies, Pseudo Amino Acid Composition (PseAAC) was designed^19^. PseAAC can consider global sequence order information and local sequence order information in a protein sequence. PseAAC uses to compute the sequence correlation factors in addition to AAC. We can formulate PseAAC using the following equation:

3 $$A=({A}_{1}, {A}_{2}, {A}_{3}, .\dots \dots .., {A}_{20+{A}_{1}})$$

where the first 20 dimensions of $A$ shows the frequency of amino acids and λ computes the correlation factors. In order to capture the local region’s information encoded in PseAAC, we extended the notion of segmentation into PseAAC and thus generated a novel descriptor (S-PseAAC).

### Feature selection approach

In feature vector, some features are effective and can improve the performance of the model. The feature selection method is used to select these effective features to enhance the performance of the proposed method. We selected the best features by employing the combination of XGB and RFE approaches. First, the XGB formulates the significant point of each feature and assigns weight to each feature. Second, the weighted sum of the scores of each feature in all boost trees is utilized to achieve the final importance score. Third, the features are arranged according to the final score. Fourth, after getting the importance ranking of features, Finally, RFE eliminates the less informative features from the feature space^27^. This process continues to N times until the required number of features is attained.

In this work, we selected 17, 73, and 36 best features from GDPC, RAAA, and S-PseAAC, respectively. These best features were concatenated to make a superset.

### eXtreme gradient boosting

XGB is a dominating classifier that was introduced by Chen and Guestrin^28^. In recent years, XGB showed shining performance in many classifications and challenging problems. XGB incorporates several novel features into gradient tree boosting notion which enhances its speed and performance. It is a scalable system almost in all scenarios and therefore wins several machine-learning-based competitions^29^. The scalability of XGB is due to several algorithmic optimizations and important features including handling sparse data with new tree learning scheme, handling instance weights in approximate tree learning using theoretically justified weighted quantile sketch procedure^30^. Distributed and parallel computing makes the learning process quicker that leads to fast model exploration^28^. More importantly, XGB applies the regularization notion in the loss function which not only avoids overfitting issues but also controls the complexity.

**Figure 2:**
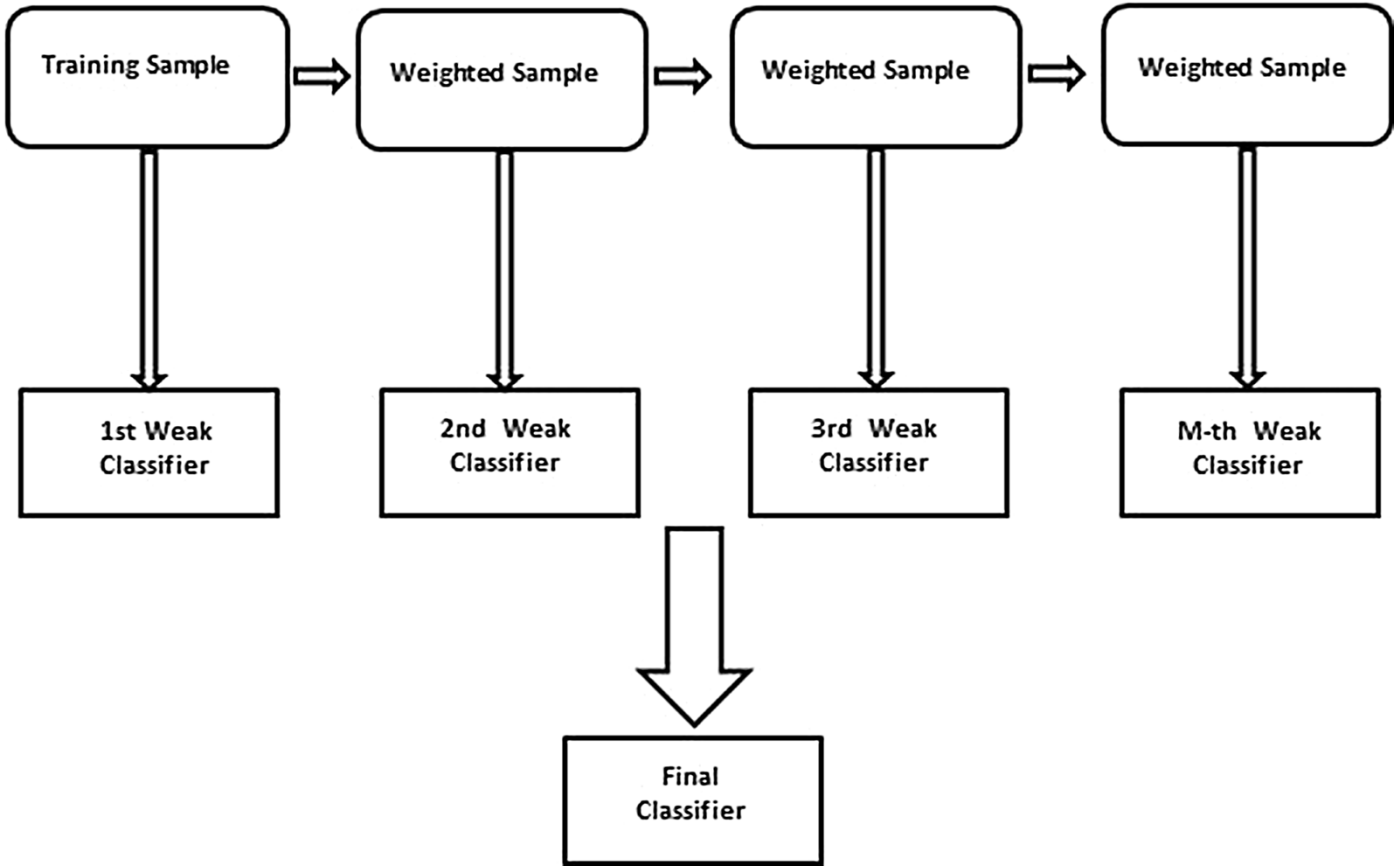
Simple architecture of XGB.

In this work, we generate competent models from several individual weak learners in an iterative way. Initially, the first model is trained by selecting samples randomly from the dataset having equal weights and equal chances to contribute in the training. Each model is tested on all samples in the dataset and the weights of the misclassified samples are updated to pick for selection in the next model training. Sequentially, several models are designed. During the testing phase, a test sample is classified according to the prediction of majority models. The working chart of the XGB is shown in Fig. 2.

We used several hyperparameters like estimator, eta, max depth, alpha, and lambda to improve the model performance. The “estimator” is used to generate the number of trees, “eta” regulates the learning rate, “max depth” controls the depth of the tree, “lambda” is used to avoid the overfitting, and “alpha” shrinks the high dimensional dataset. These hyperparameters are tuned by grid search approach. The list of these hyperparameters and their values are reported in Table 1.

**Table 1:** Hyperparameters of the proposed model.

Hyperparameter	Value
No. of estimator	500
Era	0.1
Max depth	8
lambda	1
alpha	1

### Performance evaluation

After designing a computational predictor, the performance is evaluated using different validation methods. The most employed validation schemes are jackknife and k-fold cross-validations^31–37^. However, jackknife approach has high cost and computational time^38–44^. This study implements tenfold cross-validation and five parameters i.e., accuracy (Acc), sensitivity (Sn), specificity (Sp), F-measure, and Mathew’s correlation coefficient (MCC) for examining the performance of the model.

The Acc, Sn, Sp, F-measure, and MCC can be formulated using the following equations:

4 $$\mathrm{A}cc= 1-\frac{{DP}_{-}^{+}+{DP}_{+}^{-}}{{DP}^{+}+{DP}^{-}}$$

5 $$Sn= 1-\frac{{DP}_{-}^{+}}{{DP}^{+}}$$

6 $$Sp= 1-\frac{{DP}_{+}^{-}}{{DP}^{-}}$$

7 $$MCC= \frac{1-\left(\frac{{DP}_{-}^{+}+{DP}_{+}^{-}}{{DP}^{+}+{DP}^{-}}\right)}{\sqrt{\left(1+\frac{{DP}_{-}^{+}+{DP}_{+}^{-}}{{DP}^{+}}\right) \left(1+\frac{{DP}_{-}^{+}+{DP}_{+}^{-}}{{DP}^{-}}\right)}}$$

8 $$F-measure=2*(precision*recall/precision+recall)$$

8 $$Precision=\frac{{DP}^{+}}{{DP}_{-}^{+}+{DP}^{+}}$$

9 $$Recall=\frac{{DP}^{+}}{{DP}_{+}^{-}+{DP}^{+}}$$

where ${DP}^{+}$ and ${DP}^{-}$ represent the true positive (druggable protein) and true negative (non-druggable protein), respectively. Similarly, ${DP}_{+}^{-}$ denotes the number of false negative predictions that the model incorrectly predicted as true and ${DP}_{-}^{+}$ shows the samples that the model incorrectly predicted as false that are initially labeled as true.

## Results and discussion

### Performance of classifiers before feature selection

In this work, features from dataset are captured by group dipeptide composition, reduced amino acid alphabet, and novel encoder pseudo amino acid segmentation. The feature vector of each feature descriptor is fed into three classifiers i.e., Random Forest, Extremely Randomized Tree, and eXtreme Gradient Boosting. The performance of all classifiers is evaluated with tenfold CV and summarized the results in Table 2. The ERT using RAAA secures Acc of 81.10%, Sn of 88.10%, Sp of 75.59%, F-measure of 82.84%, and MCC of 0.64. ERT enhances the performance on GDPC and S-PseAAC, and achieves 84.65% and 89.33% accuracies, respectively. The results show that both GDPC and S-PseAAC captured informative features. RF generated better performance than ERT and yielded an accuracy of 82.61% on RAAA. RF also improved the prediction results with GDPC, S-PseAAC, and All features set dimensions. Among all, RF has secured the highest results on the combination of All features set.

**Table 2:** Performance of classifiers before feature selection.

Classifier	Feature descriptor	Acc (%)	Sn (%)	Sp (%)	F-measure (%)		MCC
ERT	RAAA	81.82	88.10	75.59	82.84		0.64
	GDPC	84.65	83.04	85.92	82.67		0.68
	S-PseAAC	89.33	88.89	89.76	89.24		0.78
	All features	88.14	87.83	88.41	88.69		0.80
RF	RAAA	82.61	86.51	78.74	83.21		0.65
	GDPC	83.86	83.93	83.80	82.10		0.67
	S-PseAAC	89.72	87.30	92.13	89.43		0.79
	All features	90.12	85.22	94.20	88.69		0.80
XGB	RAAA	83.79	84.92	82.95	83.92		0.67
	GDPC	86.22	80.36	90.85	83.72		0.72
	S-PseAAC	90.51	91.27	89.76	90.55		0.81
	All features	92.09	91.30	92.75	91.30		0.84

From Table 2, we can see that XGB raises the results on all parameters i.e., Acc, Sn, Sp, F-measure, and MCC. The best results of XGB have been noted over All features set and acquired an accuracy of 92.09%. These results are not only higher than RAAA, GDPC, and S-PseAAC but also surpassed RF and ERT classifiers. Comparing the performance of individual feature extraction methods i.e., RAAA, GDPC, and S-PseAAC, it is noted that S-PseAAC generates good prediction results with all classifiers. S-PseAAC with ERT has increased the accuracies by 7.51% and 4.68% than RAAA and GDPC, respectively. Similarly, 7.11% and 5.86% higher accuracies are secured by S-PseAAC using RF than RAAA and GPDC. S-PseAAC with XGB further improved the performance and attained the highest accuracy i.e., 90.51%. It is verified by S-PseAAC that extending segmentation strategy into PseAAC is more helpful in extracting the local discriminative information and contributing greatly to the design of XGB-DrugPred model.

### Performance of classifiers after feature selection

The multi-perspective feature set extracted from different encoders may reflect decisive information. However, high dimensional feature vector may affect the performance of a model. To eliminate the redundant, noisy, and less informative features as well as reduce the computational time, we adopted XGB-RFE as feature selection algorithm. With XGB-RFE, we ranked features of each descriptor i.e., GDPC, RAAA, and S-PseAAC according to their importance. We selected 17, 73, and 36 optimal features from GDPC, RAAA, and S-PseAAC, respectively. These best features are provided to ERT, RF, and XGB machine learning algorithms for model training, validated the performance of each classifier with tenfold, and reported prediction results in Table 2. From Table 2, we can see that after applying feature selection approach, all classifiers improved the prediction performance mostly on all feature vectors. For instance, the accuracy of ERT with RAAA before feature selection algorithm is 81.82% and after applying feature selection is 82.21%. RF enhances the accuracy by 0.79% using the RAAA. XGB has attained an accuracy of 84.82% after feature selection over RAAA which is 1.03% higher than before applying feature optimization technique with same feature encoder and classifier. Similarly, the classifiers on the models of other feature vectors have shown remarkable outcomes. On S-PseAAC, the accuracies reported by ERT, RF, and XGB are 90.12%, 90.91%, and 91.70%, respectively which are higher than RAAA and GDPC descriptors. This reveals that incorporating segmentation into PseAAC can capture important local patterns. It is reported by past studies that combination of heterogeneous features set may generate better results^45^. In this connection, we combined the optimal features of all encoders and provided them to classifiers. Table 3 describes that all classifiers have achieved promising results with All feature sets. However, among all classifiers, XGB yielded 94.86% accuracy which is 2.77% higher than before feature selection on All features set. It is concluded that the selection of the best features performed a significant role in the development of the proposed model.

**Table 3:** Performance of classifiers after feature selection.

Classifier	Feature descriptor	Acc (%)	Sn (%)	Sp (%)	F-measure (%)		MCC
ERT	RAAA	82.21	84.91	79.53	82.63		0.64
	GDPC	81.10	77.44	85.12	81.10		0.62
	S-PseAAC	90.12	84.82	94.33	88.37		0.80
	All features	92.09	91.96	92.20	91.15		0.84
RF	RAAA	83.40	83.33	83.46	83.33		0.66
	GDPC	82.28	77.45	87.60	82.07		0.65
	S-PseAAC	90.91	84.85	85.73	89.20		0.81
	All features	93.28	92.86	93.62	92.44		0.86
XGB	RAAA	84.82	84.92	82.68	83.92		0.67
	GDPC	83.07	81.95	84.30	83.52		0.66
	S-PseAAC	91.70	88.39	94.33	90.41		0.83
	All features	94.86	93.75	95.74	94.17		0.89

### Comparison of the proposed model with existing methods

**Figure 3:**
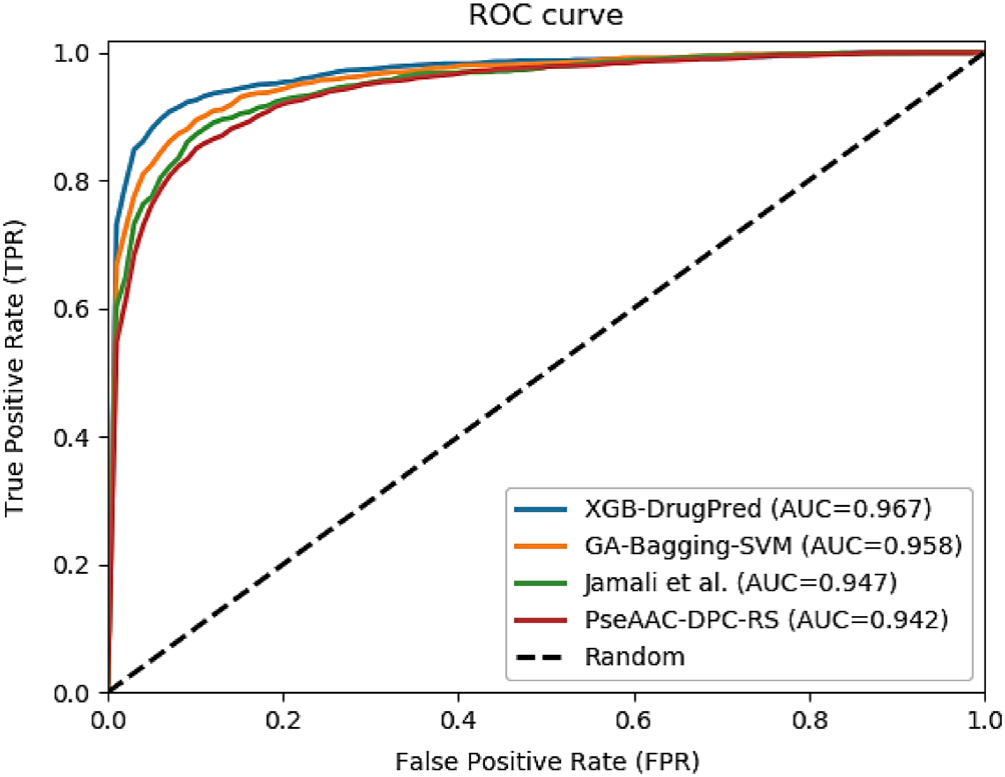
ROC curves of the proposed and existing methods.

To assess the efficacy of the proposed predictor, we compare the prediction results with existing predictors including PseAAC-DPC-RS, Jamali et al., and GA-Bagging-SVM. The accuracy, sensitivity, specificity, and MCC of the first-best predictor (GA-Bagging-SVM) are 93.78%, 92.86%, 94.45%, and 0.87, respectively while our predictor yielded 94.86% accuracy, 93.75% sensitivity, 95.74% specificity, and 0.89 MCC. Analyzing the prediction results, we can see from Table 4 that XGB-DrugPred has achieved 1.08% Acc, 0.89% Sn, 1.29% Sp, and 0.02 MCC higher than the best method. The XGB-DrugPred boosted 2.76% Acc, 0.95% Sn, 4.4% Sp, and 0.05 MCC than second-best method. Similarly, our predictor surpassed the PseAAC-DPC-RS on all evaluation parameters. After performing the comparison, it is observed that proposed predictor for prediction of druggable proteins is more effective than all existing predictors in the literature. The ROC curves and AUC values of the proposed model and the existing models have provided in Fig. 3.

**Table 4:** Comparison with existing predictors.

Predictor	Acc (%)	Sn (%)	Sp (%)	MCC
PseAAC-DPC-RS	90.98	87.88	94.11	0.82
Jamali et al	92.10	92.80	91.34	0.84
GA-Bagging-SVM	93.78	92.86	94.45	0.87
XGB-DrugPred	94.86	93.75	95.74	0.89

## Conclusion

Druggable protein prediction with experimental methods is laborious and high cost. The pharmaceutical industry employed machine learning predictors to capture properties of successful drug-targets to predict novel drugs with the same properties. In this connection, we also make efforts and developed a novel predictor for druggable proteins. This work explores the features by RAAA, GDPC, and S-PseAAC. The optimal feature selection is performed by XGB-RFE. The classification is carried out by ERT, RF, and XGB. Among these, XGB with the best feature set achieved the highest performance. The superior performance of the XGB-DrugPred is due to several reasons including the application of appropriate feature encoding methods, effective feature selection scheme, and powerful classifier. In future work, we make efforts to establish a web server for the proposed predictor that will be fruitful for academicians and researchers. More importantly, our novel predictor will be helpful to capture a more universal view of a potential target.

## Future direction

MicroRNAs (miRNAs) have been proved to be targeted by the small molecules recently, which made using small molecules to target miRNAs become a possible therapy for human diseases^46^. Therefore, it is very meaningful to investigate the relationships between small molecules and miRNAs. In this connection, several experimental and computational models have been developed and implemented to identify novel small molecule-miRNA associations^47–49^. The small molecules inhibit a specific function of a multifunctional protein and may have beneficial effect against diseases. It is reported that small molecules make up 90% of pharmaceutical drugs (such as insulin, aspirin, and antihistamines)^50^. Like druggable proteins, a kind of small molecules comprises amino acids. Thus, in addition to druggable proteins, the proposed study can predict the small molecules of drugs or the association of small molecules of drugs with miRNA using primary sequences. As small molecule-miRNA associations are significant for discovering novel drugs against many human diseases. Therefore, in future, we will try to develop computational methods for accurate prediction of small molecule-miRNA associations using effective feature extraction and selection algorithms.

## Data and material availability

In future work, we will make efforts to establish a web-server that is freely accessible for researchers and academicians. Presently, the source code and datasets are available freely at link https://github.com/wangphd0/drug.
